# A new model isolates glioblastoma clonal interactions and reveals unexpected modes for regulating motility, proliferation, and drug resistance

**DOI:** 10.1038/s41598-019-53850-7

**Published:** 2019-11-22

**Authors:** Justin B Davis, Sreshta S Krishna, Ryan Abi Jomaa, Cindy T. Duong, Virginia Espina, Lance A Liotta, Claudius Mueller

**Affiliations:** Center for Applied Proteomics and Molecular Medicine, George Mason University, 10920 George Mason Circle, Manassas, VA 20110 USA

**Keywords:** CNS cancer, Tumour heterogeneity, Collective cell migration

## Abstract

Tumor clonal heterogeneity drives treatment resistance. But robust models are lacking that permit eavesdropping on the basic interaction network of tumor clones. We developed an *in vitro*, functional model of clonal cooperation using U87MG glioblastoma cells, which isolates fundamental clonal interactions. In this model pre-labeled clones are co-cultured to track changes in their individual motility, growth, and drug resistance behavior while mixed. This highly reproducible system allowed us to address a new class of fundamental questions about clonal interactions. We demonstrate that (i) a single clone can switch off the motility of the entire multiclonal U87MG cell line in 3D culture, (ii) maintenance of clonal heterogeneity is an intrinsic and influential cancer cell property, where clones coordinate growth rates to protect slow growing clones, and (iii) two drug sensitive clones can develop resistance de novo when cooperating. Furthermore, clonal communication for these specific types of interaction did not require diffusible factors, but appears to depend on cell-cell contact. This model constitutes a straightforward but highly reliable tool for isolating the complex clonal interactions that make up the fundamental “hive mind” of the tumor. It uniquely exposes clonal interactions for future pharmacological and biochemical studies.

## Introduction

Intratumor clonal heterogeneity is a critical problem in cancer because it leads to treatment resistance^1–3^. This challenge spans the cancer landscape^4–7^, with glioblastoma considered to have one of the highest levels of clonal heterogeneity^8–11^. Current knowledge indicates that different clones with unique drug resistance profiles may exist pre-treatment^2,3,12^. Treatment then changes the clonal makeup of the tumor such that previously underrepresented, but treatment-resistant, clones take over the mass of the tumor^3^. Heterogeneous clones co-exist within the tumor for a long time, if not from the very early stages immediately following tumor initiation^13^. In this context it is important to differentiate between phenotypic heterogeneity, such as found when tumor stem-cells give rise to tumorigenic and non-tumorigenic progeny^14^, and genotypic (clonal) heterogeneity. Although the mechanisms underpinning the development of clonal heterogeneity are still under debate, the basis for maintaining distinct clonal populations in all tumor evolution models is spatial diversity within the microenvironment^2^. It is assumed that different environmental constraints allow for the expansion of the clone that is best adapted to that particular environment. However, if that were true, long-term maintenance of tumor clones with sub-optimal fitness would not exist in homogeneous environments such as *in-vitro* cell culture.

**Figure 1:**
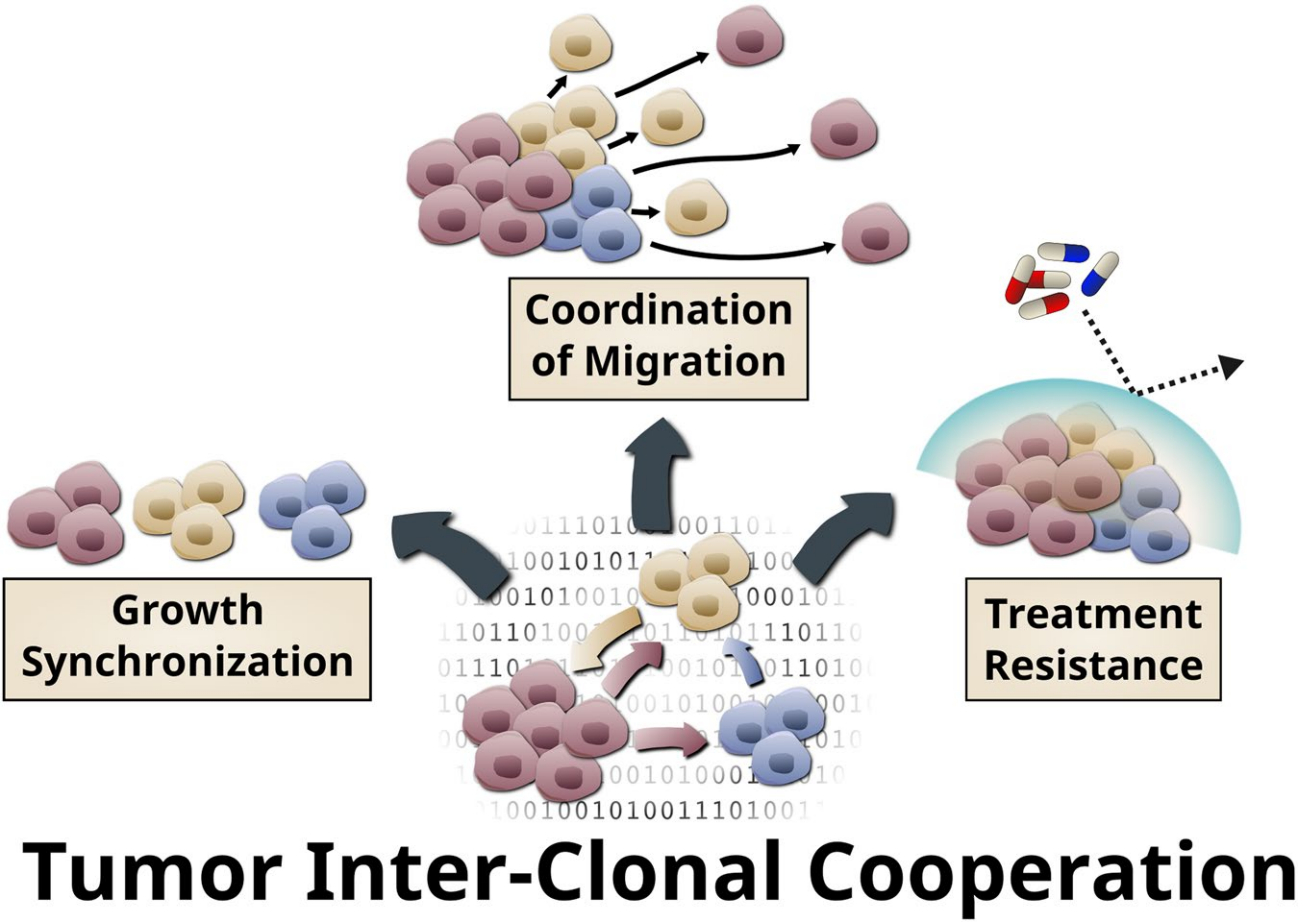
Tumor cell clones cooperate to (i) synchronize their growth rate, (ii) coordinate migration and (iii) resist treatment.

Communication is a basic principle of success for any societal community, be it macrobiological (i.e. social insect states, human communities) or microbiological (i.e. coordinated resistance to antibiotics in bacteria^15^). It is therefore sensible to assume that the interactions between clones are as multi-faceted and complex as in other biological communities, including multiple types of negative (i.e. competition, amensalims, predation, parasitism) and positive (i.e. commensalism, synergism, mutualism) interactions^16^. While our current models of tumor evolution focus on competition between clones, examples of tumor cell cooperation have been demonstrated^17–23^. In fact, clonal heterogeneity in itself has been found to be a prognostic marker associated with poor survival in a pan-cancer analysis of over 3300 tumors^24^. Interactions between tumor cells and various host cells of the tumor microenvironment have been studied extensively, but interactions between tumor cell clones remain elusive and largely unstudied. Even recent studies on tumor heterogeneity are limited to describing variable clonal phenotypes, without considering the emergence of novel properties when tumor clones interact^25–29^. We therefore set out to answer three fundamental questions that have not been addressed before: (i) Can one clone switch off the independent motility of another clone? (ii) If a fast and slow growing clone are intermixed, does the fast growing clone out-compete the slower clone, or do they cooperate to maintain constant fractions of each other. (iii) If two clones have the same drug sensitivity, can they cooperate to become drug resistant? (Fig. 1).

Because the effects of clonal interactions are very difficult to study directly, we developed an *in-vitro* model, based on U87MG glioblastoma cells, that eliminates all extrinsic variables, isolating the direct clonal cooperation or competition. Any type of cell or tissue culture model exerts clonal selection pressure on the cell population, and the decision to culture cells with or without serum will selectively inhibit or promote stem cell outgrowth. Even primary cell cultures, or xenograft models, that more closely resemble the original tumor biology, show significantly altered clonal heterogeneity^30,31^. More importantly, early primary cell culture or xenograft tumor establishment causes an active shift in the clonal landscape. Our aim in establishing our model system was to measure pure clonal interactions, without (i) any interference by the tumor microenvironment, and (ii) any impact of spacial inhomogeneities (oxygen gradient, nutrient gradient, distance to stromal cells, accessibility by immune cells, etc.) typically found in tumors. We therefore purposely chose the well established U87MG cell line, maintained in a homogeneous cell-culture environment, expecting a very stable phenotype of interactions between subclones that is maintained long-term. Using this straightforward and highly reliable model, we found a much richer fundamental clonal interaction phenotype than known before.

## Results

### U87MG subclones cluster into distinct signal protein pathway subtypes

**Figure 2:**
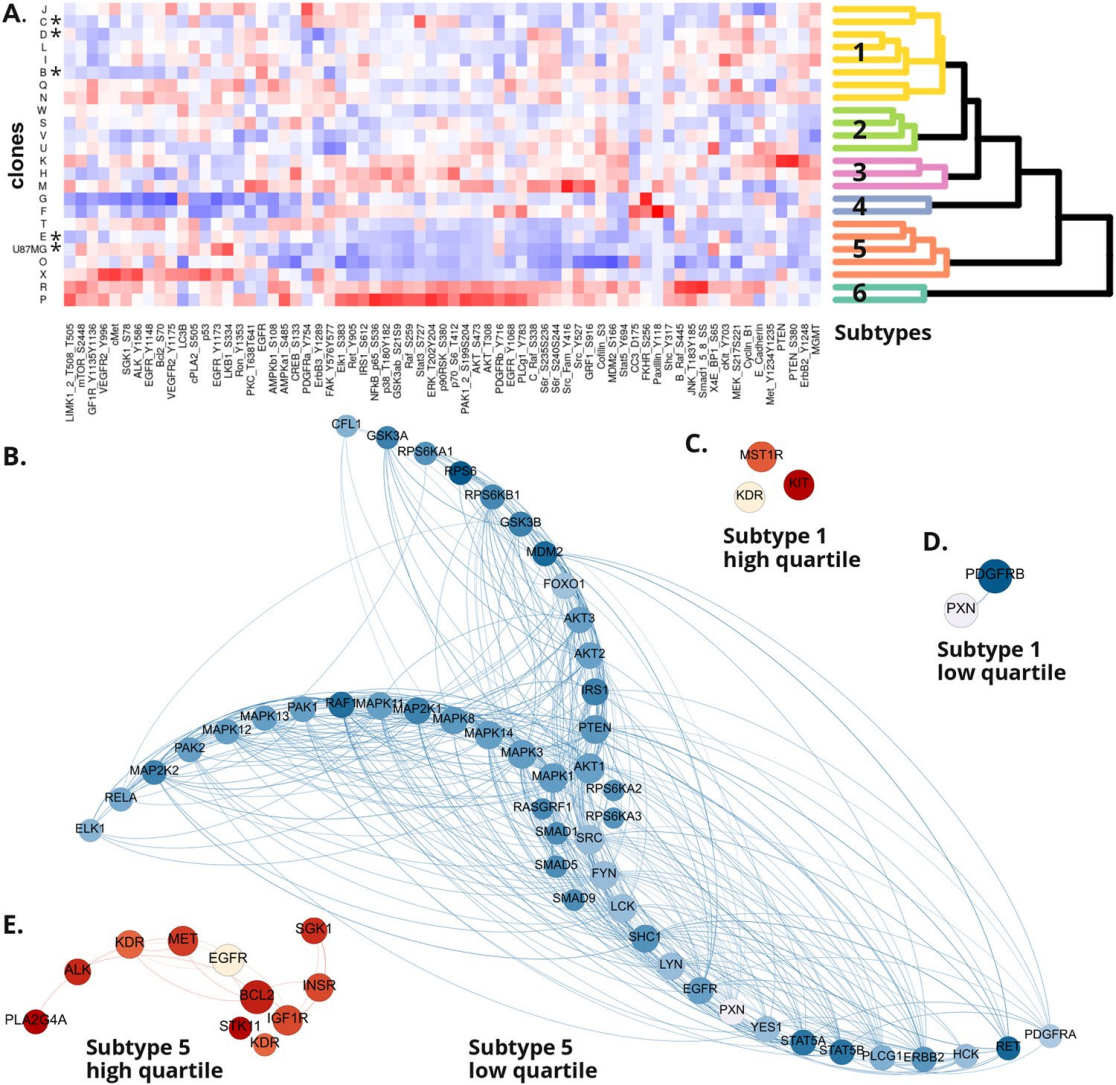
The U87MG cell line is clonally heterogeneous. (**A**) Unsupervised two-way hierarchical clustering of protein phosphorylation and abundance for 23 subclones of the U87MG cell line reveals six subtypes (*clones selected for further investigation). (**B–E**) Highest and lowest (phospho)protein abundance quartiles for subtype 1 and 5. Protein-protein interactions as predicted by STRING were plotted using Gephi, with radial arms representing separate protein interaction clusters based on modularity. Node size and order is reflecting degree (number of connections per node), while color shade corresponds to protein abundance level (proteins described by gene names as understood by STRING, red = highest quartile, blue = lowest quartile).

We picked 96 individual U87MG cells, of which 23 (24%) could be expanded into monoclonal cell lines that each maintained a fully stable observable phenotype. We then characterized the phosphorylation status and/or abundance of 68 key cell signaling proteins in the clones and the parental U87MG cell line using reverse phase protein microarrays (Fig. 2A, Supplemental Table S1). Unsupervised hierarchical 2-way clustering showed extensive diversity between the individual clones, distributed among six major subtypes (Fig. 2A). Each subtype was characterized by a distinct set of upregulated (highest quartile) and downregulated (lowest quartile) phosphoproteins involved in key cancer signaling pathways, such as PI3K-Akt signaling, ErbB signaling, MicroRNA signaling, and focal adhesion (Table 1, Fig. 2B–E, Supplemental Fig. 1).

**Table 1:** Top three KEGG enriched pathways within the highest and lowest (phospho)protein abundance quartiles for each clonal subtype.

Regulation	Subtype	% proteins measured	Pathway 1	Pathway 2	Pathway 3
**upregulated**	1	4%	Ras signaling	Rap1 signaling	Endocytosis
	2	3%	—	—	—
	3	40%	Prolactin signaling	Proteoglycans in cancer	ErbB signaling
	4	15%	Focal adhesion	Natural kill cell mediated cytotoxicity	MicroRNAs in cancer
	5	15%	PI3K-Akt signaling	Ras signaling	FoxO signaling
	6	72%	ErbB signaling	FoxO signaling	Proteoglycans in cancer
**downregulated**	1	3%	Focal adhesion	Regulation of actin skeleton	—
	2	18%	Proteoglycans in cancer	Ras signaling	Pathways in cancer
	3	7%	McroRNAs in cancer	MAPK signaling	PI3K-Akt signaling
	4	41%	PI3K-Akt signaling	FoxO signaling	Pathways in cancer
	5	47%	ErbB signaling	Prolactin signaling	T cell receptor signaling
	6	4%	Focal adhesion	Regulation of actin skeleton	Proteoglycans in cancer

### Clones show a distinct genotype

**Figure 3:**
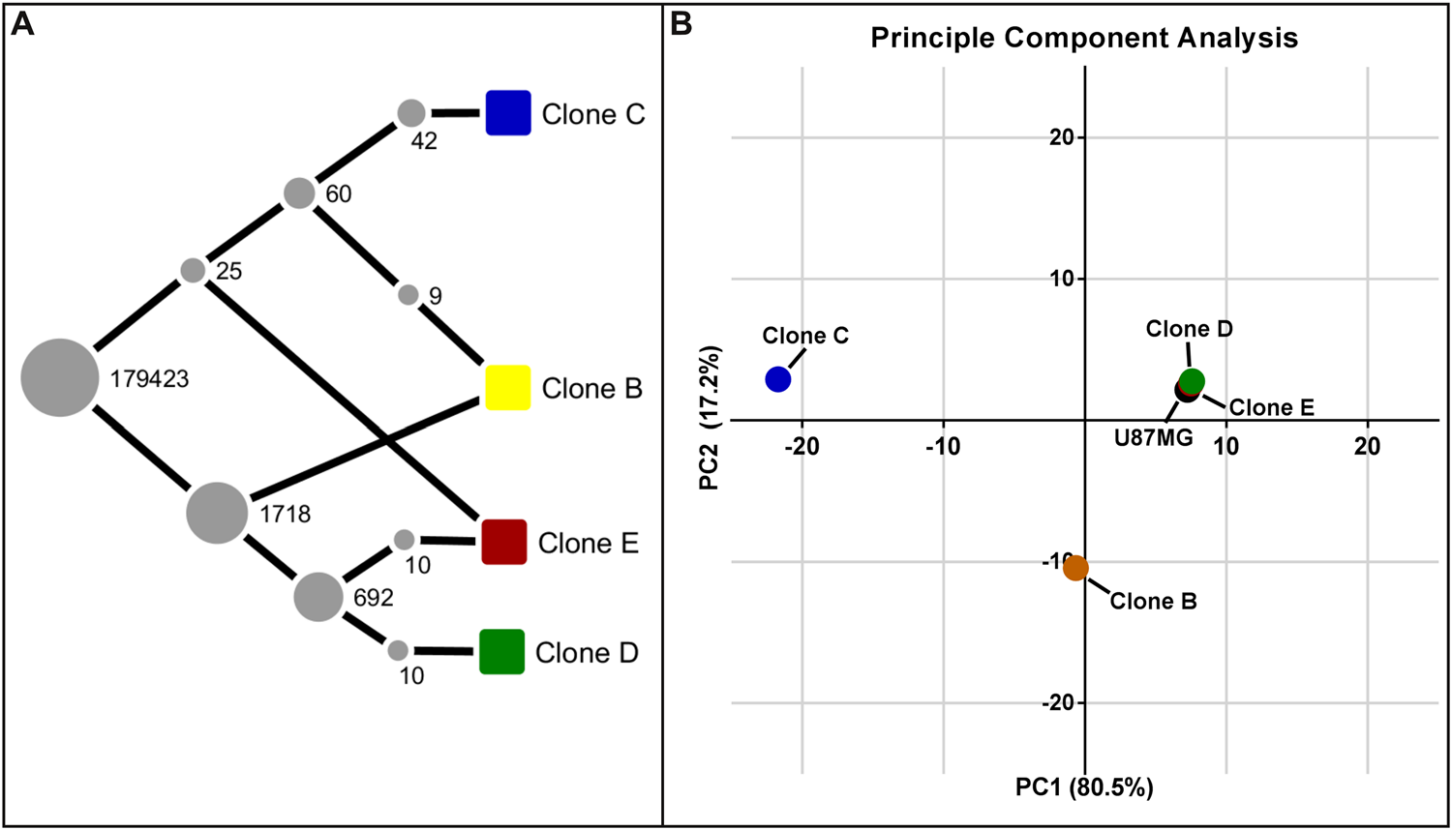
Single nucleotide polymorphism analysis reveals the evolutionary relationship between clones and the relative abundance of each within the U87MG cell line. (**A**) Phylogenetic inference using LICHEeE demonstrates a close relationship of clones **D** and **E**, while clone C shows the greatest difference in genetic phenotype to all other clones. (Nodes (grey) represent the number of SNPs shared between sets of samples and edges represent ancestry relationships). (**B**) Principal component analysis of the genotype annotations indicates that the U87MG cell line is largely composed of clones **D** and **E** as well as genetically similar clones, while clone C is likely to make up a small fraction of cells within that cell line.

Clones B, C, D, and E were chosen for further genomic and phenotypic analysis based on preliminary data indicating significantly different growth rates between these clones. We focused on the growth rate phenotype difference to determine whether clones could functionally interact to influence each other’s growth rate, migration, and treatment resistance, irrespective of their individual molecular similarities or differences. All four selected clones were authenticated as U87MG via short tandem repeat profiling (ATCC, Manassas, VA, USA) (Supplemental Table 2). This was supported by single nucleotide polymorphism analysis, which demonstrated 98.6% identity of the evaluated SNP loci between all four clones. Nonetheless, each clone presented with its own unique genotype. Phylogenetic inference identified a tight similarity between clone E and clone D. Clone C possessed the greatest difference in genotype compared with the other selected clones (Fig. 3A). While this corroborates the relational difference between clone C and E shown within the proteomic data, clones C, D, and B were previously found within the same proteomic subtype. To indicate the abundance of each respective clone within the U87MG cell line, principal component analysis was performed using the genotypes of U87MG and its four selected subclones (Fig. 3B). The U87MG genotype associated tightly with clones D and E and was distant from clone C, suggesting that the cell population within this cell line is mostly composed of clones D and E as well as other clones closely related in genotype. This data further shows that, among the four subclones evaluated, clone C is the least abundant within the U87MG cell line.

### Clones regulate each other’s migration

Previous studies have indicated that invasiveness and migration of tumor cells can benefit from cooperation between clones^19,32,33^. We therefore evaluated clonal interactions within our model in a 2D wound healing assay and a 3D spheroid assay, whereby spheroids were transplanted onto cell culture treated plastic to permit unguided cellular migration radially outward from the spheroid. Both assays were performed without providing any chemotaxis cues in order to evaluate unguided migration speed, and controls were included to control for total number of cells present in each well/spheroid and number of cells per clone.

**Figure 4:**
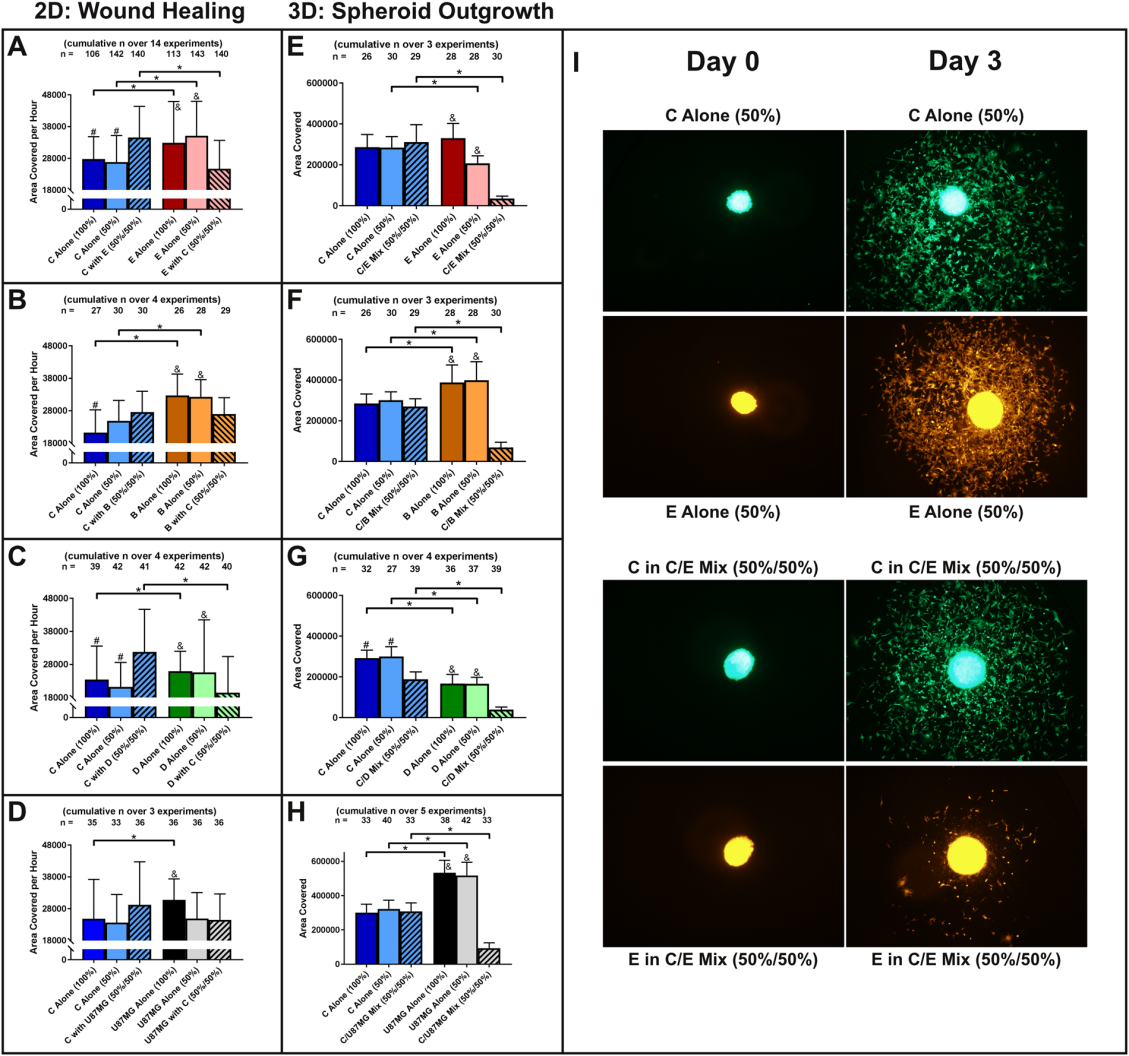
Tumor cell clones coordinate their migration. (**A–D**) In a 2D wound healing assay, clone C inhibits migration of any other clone present while increasing its own migration. (E–H) 3D spheroid outgrowth onto cell culture treated plastic demonstrates significant inhibition of migration of any clone co-localized with clone C. (**I**) Representative images of spheroid outgrowth of clones C and E either alone (top panel) or mixed (bottom panel). (*p < 0.05 for indicated pairs; ^#,&^p < 0.05 versus respective clone in mix).

In both, the 2D and 3D assays, clone C significantly inhibited clone E migration (up to 8.3 fold, p < 1*10^−14^), while increasing its migration rate when co-cultured with clone E in the 2D assay (1.3 fold, p < 1*10^−10^) (Fig. 4A,E,I). Likewise, co-culturing clones C and B resulted in a significant inhibition of clone B migration in the 2D and the 3D assay (up to 5.8 fold, p < 1*10^−40^). This caused clone C to either synchronize (2D) or significantly outperform (3D) clone B migration, even though clone C showed a significantly slower migration rate than clone B when cultured separately (2D: up to 1.7 fold, p < 1*10^−4^, 3D: 1.3 fold, p < 1*10^−6^) (Fig. 4B,F). Similarly, clone C inhibited clone D migration in both assays. However, in contrast to co-cultures of clone C with clone E or B, clone D reciprocally inhibited clone C migration, albeit to a lesser degree (clone C: 1.6 fold reduction, p < 1*10^−11^, clone D: 4.3 fold reduction, p < 1*10^−20^) (Fig. 4C,G).

Since clone C was able to inhibit the migration of any clone it was in contact with, we then compared the impact of clone C on the parental U87MG cell line. Although clone C cells are part of that cell line, and thereby confound the results with unknowable impact, we wanted to determine if clone C was able to inhibit the complete mix of U87MG clones when present at a large fraction of the total cell population. In the wound healing assay, no significant change in migration rate was observed that could not be explained by seeding density (Fig. 4D, see high and low seeding density controls). However, in the 3D spheroid model, the same pattern emerged that was seen in other co-cultures of clone C. Separately, U87MG cells migrated faster than clone C cells (1.8 fold, p < 1*10^−18^, Fig. 4H). But when cultured together, the migration rate of U87MG cells was significantly inhibited (5.7 fold, p < 1*10^−20^), while clone C migration rate remained unchanged. This resulted in a significantly increased migration rate for clone C over U87MG cells in the mix (3.3 fold, p < 1*10^−19^, Fig. 4H).

### Individual clones actively synchronize their growth rate when grown together

**Figure 5:**
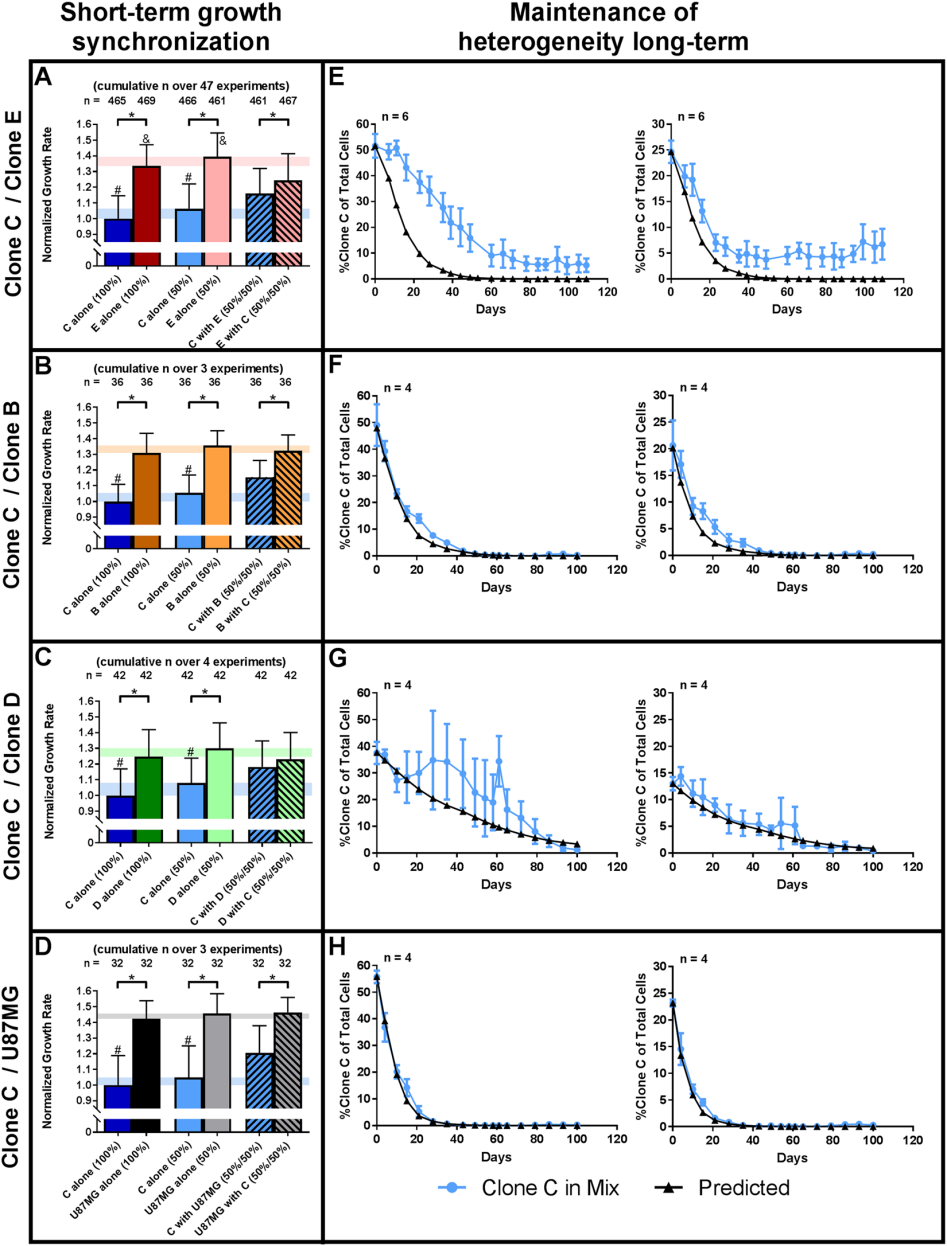
Tumor cell subclones coordinate each other’s growth rate. (**A–D**) Within six days, clone C increases its growth rate in the presence of faster growing clones. Simultaneously, clone E reduces its growth rate to synchronize growth with clone C. In contrast, clones B, D, and the U87MG cell line maintain a constant growth rate, regardless of the presence or absence of clone C (n = 32 to 469, cumulative over a maximum of 47 experiments). (**E–H**) In long-term cell culture (minimum of 100 days), slow growing clone C is ultimately maintained at a constant fraction of 5.9% of total cells when mixed with fast growing clone E, regardless of the initial seeding ratio. When mixed with clones B, D, or the U87MG cell line, the ratio of clone C largely follows prediction and is ultimately outcompeted. (*p < 0.05 for indicated pairs; ^#,&^p < 0.05 versus respective clone in mix; long-term co-culture was only performed one time with 4–6 replicates due to the extended time frame of the experiment).

Preliminary data indicated that the growth rate of clone C was significantly lower than the parental U87MG cell line. We therefore sought to understand how clones with different proliferation rates can coexist in a homogeneous cell culture environment. Clone C showed a significantly reduced growth rate compared to the parental U87MG cell line (1.4 fold, p < 1*10^−13^), clone B (1.3 fold, p < 1*10^−17^), D (1.2 fold, p < 1*10^−7^), and E (1.3 fold, p < 1*10^−118^) (Fig. 5A–D). Although the observed differences in growth rate may seem small, the impact is very significant when considering growth over time (see predicted clonal elimination, Fig. 5H). Within six days of co-culturing clone C cells with U87MG cells at a 1:1 ratio clone C significantly increased its growth rate (1.2 fold, p < 1*10^−3^), while the growth rate of U87MG remained constant (Fig. 5D). In long-term cell culture (100 days), clone C was eliminated within the U87MG background, as predicted, within six passages (Fig. 5H). Because these results were confounded by an unknown fraction of clone C cells that are naturally part of the U87MG cell line, we decided to mix clone C with a monoclonal population of clone E cells. Clone E had a growth rate very similar to the U87MG cell line and was the closest related clone to U87MG cells in our proteomic and genotypic analyses (Figs 2A and 3B). Again, clone C increased its growth rate in the presence of clone E (1.1 fold, p < 1*10^−18^, Fig. 5A). However, clone E simultaneously reduced its growth rate to achieve near growth synchronization between both clones (1.1 fold, p < 1*10^−42^). This effect was highly reproducible over 47 experiments with a cumulative n of up to 469. In long-term, mixed clone C + E cell culture clone C was not eliminated as mathematically predicted. Instead, the fraction of clone C dropped initially and was then stably maintained at 5.9% of total cell number (Fig. 5E). This was largely independent of the initial seeding ratio of clones C and E (5.4% at 25:75 seeding ratio). Mixing clone C with clone B or clone D resulted in increased temporary growth of clone C with no change in clone B or clone D growth rate (1.1 fold, p < 1*10^−2^, Fig. 5B,C). During long-term co-culture, the elimination rate of clone C largely followed the predicted outcome with final elimination of clone C (Fig. 5F,G).

### Clonal cooperation results in treatment resistance

The contribution of tumor heterogeneity to treatment resistance has largely been perceived as providing resistant clones that survive treatment and aggressively take over once other clones are eliminated. In contrast, we wanted to determine if cooperation between clones itself provided an overall benefit to treatment resistance. All tested clones were largely resistant to temozolomide, the current FDA-approved chemotherapeutic drug for glioblastoma treatment (data not shown). We therefore chose two other well established chemotherapy agents, docetaxel and cisplatin, that target distinct molecular mechanisms within the cell. While both have had limited utility for treatment in glioblastoma due to their low penetrance into the brain, recent studies have revisited their usefulness in combination with blood-brain-barrier penetrating nanoparticles^34–36^.

Two overlapping effects had to be considered when measuring treatment resistance in this model: (i) the actual susceptibility to treatment, and (ii) the previously demonstrated change in growth rate when clones grow in mixed-clone culture. Following, the combined effect of growth rate change and treatment resistance will be labeled as treatment “response”.

**Figure 6:**
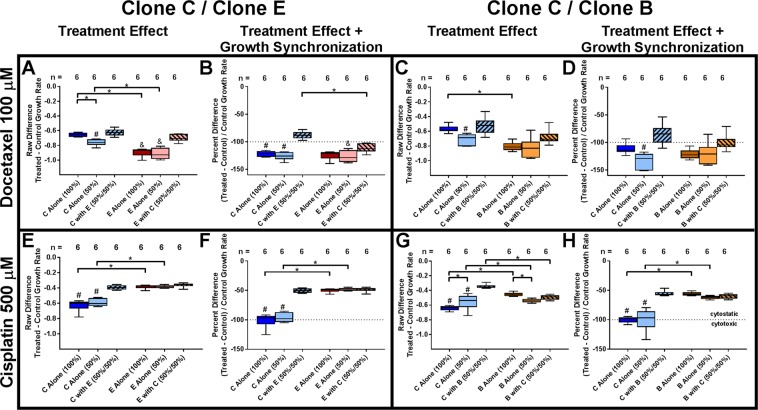
Tumor cell clones cooperate to resist treatment. (**A–D**) Cooperation between clone C and clone E leads to treatment resistance against docetaxel. (**E–H**) Clone C is significantly more resistant to treatment with cisplatin when cooperating with clone E or B, while neither clone E or B benefit from this cooperation against cisplatin treatment. (**A,C,E,G**)=raw effect of drug treatment, disregarding differences in clonal growth rate and the change in growth rate that is observed when clones are mixed; (**B,D,F,H**)=adjusted effect that accounts for clonal growth rates. (*p < 0.05 for indicated pairs; ^#,&^p < 0.05 versus respective clone in mix; each experiment was repeated at least three times, representative experiment shown).

Clone E showed a higher susceptibility to 100 μM docetaxel than clone C when grown alone (1.4 fold, p < 1*10^−6^; Fig. 6A). When both clones were cultured together, clone E significantly increased its resistance (1.4 fold, p < 1*10^−5^), which lead to synchronized susceptibility levels between both clones. Accounting for the different growth rates of clones C and E, both showed equal response to docetaxel when cultured separately, with clone C significantly increasing resistance in co-culture from a cytotoxic to a cytostatic response (1.4 fold, p < 1*10^−6^; Fig. 6B).

Clone C showed a 1.6 fold higher susceptibility to 500 μM cisplatin than clone E when grown separately (p < 1*10^−7^; Fig. 6E). In co-culture, clone C developed resistance to cisplatin equal to the resistance seen in clone E (1.6 fold, p < 1*10^−7^). This effect was exacerbated when considering the change in clonal growth rates when separate versus mixed, whereby clone E showed 2 fold higher resistance to cisplatin than clone C when cultured separately (p < 1*10^−11^), while clone C increased its resistance to the level of clone E in co-culture (2.0 fold increase, p < 1*10^−12^ Fig. 6F).

Co-culture of clones C and B did not result in a significant increase in docetaxel resistance for either clone independent of the expected change in growth rate (Fig. 6C,D). However, clone C significantly increased its resistance to cisplatin in co-culture with clone B (1.8 fold increase, p < 1*10^−11^; Fig. 6G,H).

### Clonal cooperation is contact mediated

To understand the mechanism of communication between clones we first screened growth synchronization of clones C and E under the influence of several inhibitors that target classical cell-cell communication mechanisms, including: cambinol (exosome release inhibitor), carbonoxolone (gap junction inhibitor), dibenzazepine (Notch/APPL pathway secretase inhibitor), IWP2 (Wnt signaling inhibitor), verteporfin (Hippo signaling inhibitor), napabucasin and HO-3867 (Stat3 signaling inhibitors). None inhibited interclonal cooperation (data not shown).

**Figure 7:**
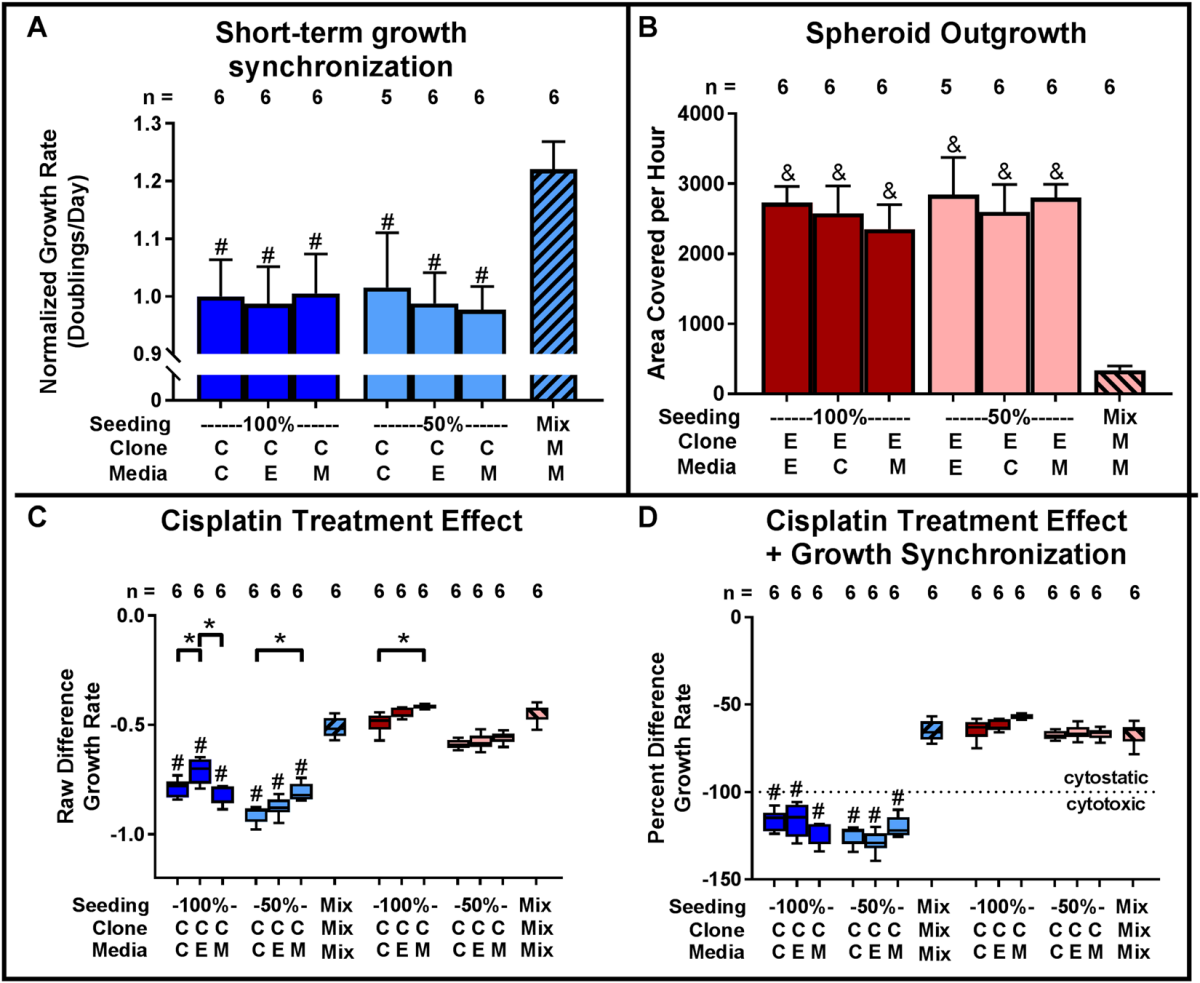
Interaction between tumor cell clones is contact mediated. (**A**) Treatment with conditioned media does not impact clone C growth. (**B**) Conditioned media does not inhibit clone E 3D spheroid outgrowth onto cell culture treated plastic. (**C**) Conditioned media does not induce treatment resistance in clone C. (*p < 0.05 for indicated pairs; ^#,&^p < 0.05 versus respective clone in mix; clone/media: C = clone C, E = clone E, M = 1:1 mix of clones C and E; each experiment was repeated at least three times, representative experiment shown).

Next, we studied the impact of conditioned media from same clone, other clone, and mixed clone cultures. Conditioned media had no effect on growth rate (Fig. 7A), migration (Fig. 7B), or treatment response (Fig. 7D). A minor increase of treatment resistance was found for clone C when adding clone E conditioned media (high seeding density) or mixed clone media (low seeding density) (Fig. 7C). Similarly, mixed clone media caused a slight increase in treatment resistance for clone E (high seeding density) (Fig. 7C). However, these effects were very small, inconsistent between seeding densities, and within the expected noise of the biological system.

Together, conditioned media from either individual clones or mixed-clone cultures did not significantly impact any of the clonal interactions observed here. This supports our hypothesis that clonal communication in this model is cell-cell contact dependent and thus opens the intriguing possibility that tumor cell clones in glioblastoma interact uniquely based on clonal identity.

## Discussion

The objective of this study was to develop a highly reproducible cell culture model that permits answering three fundamental questions: (i) can one clone inhibit the motility of another clone, (ii) can a fast and a slow growing clone co-exist long-term, (iii) and can two drug sensitive clones develop resistance de novo by cooperating together. To be able to observe phenotypes that emerge purely when clones interact required reducing variables, such as spatial inhomogeneities within the tumor (oxygen gradient, nutrient gradient, distance to stromal cells, accessibility by immune cells, etc.), and host cell influences from the tumor microenvironment. It further required a stable mix of clonal fractions that maintained the interacting phenotype long-term. Because xenograft tumors are spatially heterogeneous, and xenografts as well as cultured primary tumor cells show an active shift in the clonal landscape during establishment, we investigated basic clonal interactions in the well-established U87MG cell line. Within this cell line, any phenotype of clonal interactions could be expected to be stable and maintained long-term. In addition, the homogeneous cell-culture environment ensured that no clonal selection pressure existed due to spatial inhomogeneities. We further chose to culture cells in serum-containing media, thereby not promoting selective outgrowth of stem cells. This choice of model system imposes a limitation on the current study. The cell-cell interactions and environmental cues and constraints within an *in vivo* tumor are significantly more complex than can be captured in this model. Future studies will have to apply the foundational principles of clonal interactions reported here to models that more closely resemble *in vivo* tumor biology (i.e. short-term primary cell culture, xenografts, etc.). However, our study has shown that clonal interaction, even in the absence of host influences, can be a fundamental driver of cell behavior. It is therefore critical that we understand the basic principles that govern clonal interactions. The model system introduced here has proved to be highly consistent over time, with stable properties of the individual clones over many passages (>60), and in up to 47 replicate experiments with 469 cumulative replicate samples.

While the lineage of U87MG cells used in this study has recently been challenged^37^, it has been reconfirmed to be of human glioblastoma origin^37^. Glioblastoma is considered to have one of the highest levels of heterogeneity^8^, and the stability of such clonal heterogeneity is puzzling. A small difference in proliferation or death rate between clones should rapidly lead to the dominance of one clone within the tumor population. Selected studies have demonstrated that individual clones can release factors that support the growth of other clones^17,20,22,23^. In contrast, we demonstrate, for the first time, that clones can dynamically adjusted their growth rates positively and negatively, independent of released factors. The particular growth rate adjustment was dependent on the specific pair of clones that was interacting, and was even able to maintain constant clonal fractions long-term. Furthermore, this active maintenance of fast and slow growing clones side by side happened in a homogeneous cell culture environment, without giving clones a customized niche as they might find within the complex tumor microenvironment. This raises the intriguing possibility that maintaining clonal heterogeneity is an intrinsic property, built into cancer cell populations, independent of extrinsic influences.

Tumor cell invasion and metastasis have been found to benefit from clonal cooperation^18^. In particular the idea of “collective invasion”, where leader and follower cells of different invasive potential cooperate to increase overall invasion, is intriguing^19,32^. Our model indicates, that the repertoire of clonal interactions that impact cell motility are much richer than anticipated. We found that a single clone was able to completely switch off motility of other clones it was intermixed with, independent of chemoattractants or co-migratory behavior. Thus, while eliminating the symbiotic partnership between clones may seem intuitive to decrease tumor cell invasion, our model demonstrates for the first time that removal of a repressor clone may dramatically increase cancer cell motility.

Treatment resistance is thought to be driven by either: (i) innate drug resistance of pre-existing clones that take over once other clones have been eliminated^2,3,12^, (ii) newly acquired mutations during treatment that confer resistance, or (iii) drug resistant clones helping neighboring sensitive cells^21^. Here, for the first time, we demonstrate a new model of drug resistance, where two clones that are equally drug sensitive develop resistance when cooperating. Interestingly, this behavior was drug specific. While docetaxel resistance appeared after synergistic cooperation, cisplatin resistance was conferred from a resistant clone to a sensitive clone. In fact, taken together, our model demonstrates a much richer, and more complex, fundamental interaction network between clones than has been considered before. We found examples of amensalims (unidirectional competition between clones when migrating and during growth), mutualism (development of drug resistance not present in either clone alone), and commensalism (one clone benefiting from another clone’s drug resistance, and a slower growing clone increasing its growth rate in the presence of a faster growing clone). While it has been predicted that clonal interactions will be multi-faceted^16,38^, to our knowledge, this is the first time this diversity of positive and negative clonal interactions has been demonstrated within a single model.

Previous studies have demonstrated soluble factors, such as interleukins and growth factors, as mechanism of communication between clones^17,21–23^. We found that media fetal bovine serum content, which is a rich resource of growth factors and can shift the cost/benefit ratio for growth factor producing and growth factor dependent clones^23^, did not influence clonal cooperation during growth (data not shown). Likewise, conditioned media from either individual clones or mixed-clone cultures did not impact any of the clonal interactions observed here. This indicates direct cell-cell contact as mechanism of interaction and opens the intriguing possibility that clones can interact specifically depending on the clonal identity of the interaction partners, similar to immune cell interactions. This is supported by our finding that clonal interactions demonstrate a high level of complexity and differ depending on the individual clones participating.

We have developed a highly reproducible model of clonal interactions with stable, emergent properties, that facilitates future studies on controlling the “hive mind” of cancer cells. Using this model we have demonstrated that maintaining clonal heterogeneity is an intrinsic property, embedded into the cancer genome, even in stable cell lines within a homogeneous environment. Furthermore, this model has shown that drug resistance can be a direct result of clonal cooperation even if no individual clone is drug resistant. This supports the development of treatments that control the communication between cancer cell clones. Currently, no therapy exists that targets the cooperation of clones or specifically eliminates the driver clones that actively maintain tumor homeostasis^39^. But our results also caution against a simplistic view of targeting clonal interactions. While next generation cancer treatment must consider clonal cooperation, the downstream effects of eliminating such cooperation, or destroying driver clones, may actually be deleterious. In fact, a recent study has demonstrated the formation of a mosaic landscape of clones carrying cancer-driver mutations within the esophagus during normal aging^40^. Ecological interactions between these clones keep them in check and prohibit the formation of cancerous lesions. Our model supports this view of a complex ecosystem of clones, whereby the amalgamation of positive and negative interactions determine individual tumor properties.

It is also important to highlight, that the phenomena described here are based on the interaction of clonal populations, not individual cells. While the autocrine and paracrine cross-talk between cancer cells is undisputed, only the interactions between distinct clonal populations were able to give rise to the growth, motility, and resistance phenomena observed here.

The model introduced here provides a straightforward and powerful tool to study functional clonal cooperation and competition at a very basic level, independent of host influences. Using this tool, we have demonstrated that clonal interactions are an intrinsic and influential cancer cell property. This warrants future studies that will evaluate the impact of these clonal interactions *in vivo*, as well as determine, on a molecular level, how clones in this model system interact and exert their control over each other. Straightforward models, such as the one introduced here, can provide valuable input by enabling the isolation and exposure of these interactions for future biochemical and pharmacologic studies. This will facilitate determining how cancer cells identify and differentiate between neighboring clones and help target the mechanisms that drive the phenotypic changes that arise out of clonal interaction.

## Materials and Methods

### Establishment of clones

U87MG glioblastoma cells (ATCC, Manassas, VA, USA) were grown in MEMα media containing 10% fetal bovine serum (FBS) at 37 °C in the presence of 5% CO_2_.

Cells were detached using 0.25% Trypsin (Thermo Fisher Scientific, Waltham, MA, USA) and individual cells (n = 96) were picked at random under microscopic guidance and transferred to individual wells of a 96-well plate. This well-size was chosen because it allows for an early development of a critical mass of cells, which increases the success rate of single cell-derived monoclonal cultures. Following successful outgrowth to full monoclonal subcultures (23 of 96), selected clones were stably transfected with lentiviral particles (GenTarget, San Diego, CA, USA) to express green fluorescent protein (GFP) or red fluorescent protein (RFP). Transfected cells were not selected for target gene expression levels beyond puromycin resistance screening. Thus, transfected clones are monoclonal in terms of their single cell origin from the U87MG cell line, but polyclonal as related to the transfection with GFP or RFP. This averages out the impact that random genomic integration of the transfected gene has on the overall cell population, leading to a better preservation of the original, pre-transfection, phenotype of each clone.

Clones B, C, D, and E were authenticated using Short Tandem Repeat (STR) analysis by the American Type Culture Collection (ATCC, Manassas, VA, USA) as described in the 2012 ANSI Standard (ASN-0002). Profiling results for Clones B, C, D, and E were identical at the core loci and returned a 93% match to U87MG (Supplemental Table 2). The clones differed from the reference profile at amelogenin, not matching the Y allele present at the amelogenin locus for the database profile for U87MG. However, STR profiling, Y-chromosome painting, and Q-band assay confirmed that the clones were male in origin.

### Growth rate and toxicity assay

Clones were seeded as 1:1 mixtures of 1250 cells per clone into 96-well plate wells. To control for cells per clone as well as total cells present per well, single-clone controls were seeded at 1250 cells and 2500 cells per well. To measure toxicity, the culture medium was replaced with complete growth medium containing 100 μM Docetaxel (Selleckchem, Houston, TX, USA) or 500 μM Cisplatin (BioVision, San Francisco, CA, USA) 72 hours after seeding. Cells were imaged using a fluorescent microscope (Olympus IX51, Olympus, Center Valley, PA, USA) after seeding and again after six days of growth. Cells were counted using ImageJ and growth rates or toxicity calculated (Eq. (1)).

1 $${\rm{cell}}\,{\rm{doublings}}/{\rm{day}}=\,\log \,2({{\rm{CellCount}}}_{{\rm{final}}}/{{\rm{CellCount}}}_{{\rm{original}}})/6\,{\rm{days}}$$

Data was represented as cell doublings/day instead of standard growth curves to enable the differentiation between cytostatic and cytotoxic treatment response (Supplemental Fig. 2). For long-term growth evaluation media was exchanged two times per week and cells maintained at sub-confluence by regularly trypsinizing and removing 90% of cells from the culture for a minimum of 100 days. Predicted long-term cell growth was calculated as follows (Eq. (2)):

2 $${{\rm{CellCount}}}_{{\rm{final}}}={{\rm{CellCount}}}_{{\rm{original}}}\ast {({\rm{growth}}\_{\rm{rate}}\_{\rm{per}}\_{\rm{hour}}\ast {\rm{hours}})}^{2}$$

### Wound healing assay

Clones were seeded at 1250 cells per clone (mixed) or 1250 cells and 2500 cells per clone (alone) into wells of a 96-well plate. Following growth for six days, cell monolayers were scratched using a pipette tip and gaps were imaged immediately after scratching as well as after four hours of incubation at 37 °C in 5% CO_2_. Cell migration into the scratch was quantified using the ImageJ plugin MRI Wound Healing Tool (Volker Baecker, Montpellier RIO Imaging).

### 3D spheroid assay

Tumor cell spheroids were created in agarose coated wells^41^. In short, wells of a 96-well plate were coated with 65 μl of 1.5% agarose in serum free media. After cooling, spheroids were seeded at 1250 cells per clone (mixed) or 1250 and 2500 cells per clone (alone) into each well and incubated at 37 °C in 5% CO_2_ for three days. Spheroids were transplanted into non-agarose-coated wells with fresh media to permit unguided cellular migration out of the spheroid and imaged before and after incubation for three days at 37 °C in 5% CO_2_. Cell migration out of the spheroid was measured using ImageJ by quantifying the total area outside of the spheroid covered by cells.

### Conditioned medium assays

Clones were seeded as previously described, but with 2x the volume of complete growth medium (200 μL). After 72 hours, one half of the culture medium was replaced with fresh complete growth medium. The remaining half was replaced with 72-hour conditioned media from the same clone, a different clone, or a 1:1 mixed clone culture. To keep the amount of released factors between co-culture and conditioned media experiments equal, recipient and donor cells of conditioned media were seeded at the same cell concentration (2500 cells per well).

### Reverse phase protein microarrays (RPPA)

RPPA were printed and stained as previously described^42^. In short, cells were lysed in a 10% (v/v) Tris(2-carboxyethyl)phosphine (TCEP; Pierce, Rockford, IL) in Tissue Protein Extraction Reagent (T-PER, Pierce)/Tris-glycine 2X SDS buffer (Thermo Fisher Scientific, Waltham, MA, USA) solution and stored at −80 °C before printing. Lysates were printed on ONCYTE Avid nitrocellulose slides (Grace Bio-Labs, Bend, OR, USA) using an Aushon 2470 arrayer (Quanterix, Lexington, MA, USA) equipped with 185 μm pins and stored dessicated at −20 °C before staining. Immunostaining was performed using a Dako Autostainer according to manufacturer’s instructions (CSA kit, Dako). All antibodies used (Supplementary Table S1) were extensively validated for single band, appropriate molecular weight specificity by Western blotting prior to application for RPPA. Total protein content per spot was determined using a Sypro Ruby protein stain (Thermo Fisher Scientific). Spot raw analysis data were acquired using ImageQuant 5.2 (Molecular Dynamics) and post-processed using the Reverse Phase Protein Microarray Analysis Suite Excel Macro (developed in-house^43^).

### Analysis of single-nucleotide polymorphisms

DNA was extracted and purified using the QIAmp DNA Mini Kit (Qiagen, Valencia, CA, USA). DNA was amplified, fragmented, precipitated, re-suspended, and hybridized to CytoSNP-12 beadchips (Illumina, Inc.). Following single-base extension and DNA staining, microarrays were analyzed on an Illumina BeadStation 500 GX laser scanner. Raw fluorescence data was processed into genotypic data using Illumina GenomeStudio 2.0 software. GenomeStudio 2.0 determined minor allele frequency and annotated genotypes which were converted to a symmetrical trinary scoring format as an input for minor allele frequency. LICHEeE was used to construct a lineage tree for Clone B, Clone C, Clone D, and Clone E^44^, and the output data visualized using Cytoscape.

### Statistical analysis

Two-way unsupervised hierarchical clustering analysis was prepared using R^45^, with clonal subtype identification according to the dendrogram. Mean comparisons of growth rate, migration, and treatment response were conducted in R using Wilcoxon rank-sum or Student’s t test, depending on data normality and number of replicates. Protein interaction data for radial protein cluster graphs (Fig. 2B–E) were obtained from STRING (http://string-db.org)^46^. Proteins were grouped into quartiles of expression for each clonal subtype. The proteins from the highest and the lowest quartiles were uploaded to STRING as independent groups and the resulting node and edges data from STRING then transferred to Gephi 0.9.2^47^. The number of gene names obtained from STRING (Fig. 2B–E) and protein names from RPPA (Fig. 2A) per endpoint can differ due to assessing multiple phosphorylation sites per protein and obtaining multiple gene names per protein. To limit the impact of false positive protein-protein interaction predictions, data were filtered for a minimum STRING “combined score” of 0.5.

Principal component analysis was performed using genotype annotations from GenomeStudio 2.0 for U87MG and clones B, C, D, E. Genotypes were converted into a symmetrical trinary scoring format and individual PCA coordinates were obtained using the FactoMineR package^48^. The coordinates for the first two principal components (PC1 and PC2) were plotted using GraphPad Prism 7.01 (GraphPad Software Inc.). All bar graphs and line graphs were prepared using GraphPad Prism 7.01. *p* < 0.05 was chosen to indicate significance.

## Supplementary information

Supplementary Dataset 1

## Data Availability

Clones, protocols and raw data are available upon request.

## References

[1] van Niekerk G, Davids LM, Hattingh SM, Engelbrecht A-M (2017). Cancer stem cells: A product of clonal evolution?. Int. J. Cancer.

[2] Schmitt MW, Prindle MJ, Loeb LA (2012). Implications of genetic heterogeneity in cancer. Ann. N. Y. Acad. Sci..

[3] Kreso A (2013). Variable clonal repopulation dynamics influence chemotherapy response in colorectal cancer. Science.

[4] Kim C (2018). Chemoresistance Evolution in Triple-Negative Breast Cancer Delineated by Single-Cell Sequencing. Cell.

[5] Piotrowska, Z. *et al*. Heterogeneity and Coexistence of T790M and T790 Wild-Type Resistant Subclones Drive Mixed Response to Third-Generation Epidermal Growth Factor Receptor Inhibitors in Lung Cancer. *JCO Precis. Oncol*., **2018** (2018). 10.1200/PO.17.00263 PMC6097183 30123863

[6] Saeed Khalid, Ojamies Poojitha, Pellinen Teijo, Eldfors Samuli, Turkki Riku, Lundin Johan, Järvinen Petrus, Nisen Harry, Taari Kimmo, af Hällström Taija M., Rannikko Antti, Mirtti Tuomas, Kallioniemi Olli, Östling Päivi (2018). Clonal heterogeneity influences drug responsiveness in renal cancer assessed by ex vivo drug testing of multiple patient-derived cancer cells. International Journal of Cancer.

[7] Osuka S, Van Meir EG (2017). Overcoming therapeutic resistance in glioblastoma: the way forward. J. Clin. Invest..

[8] McGranahan N, Swanton C (2017). Clonal Heterogeneity and Tumor Evolution: Past, Present, and the Future. Cell.

[9] Johnson BE (2014). Mutational analysis reveals the origin and therapy-driven evolution of recurrent glioma. Science.

[10] Kim H (2015). Whole-genome and multisector exome sequencing of primary and post-treatment glioblastoma reveals patterns of tumor evolution. Genome Res..

[11] Wang J (2016). Clonal evolution of glioblastoma under therapy. Nat. Genet..

[12] Roche-Lestienne C (2002). Several types of mutations of the Abl gene can be found in chronic myeloid leukemia patients resistant to STI571, and they can pre-exist to the onset of treatment. Blood.

[13] Davis A, Gao R, Navin N (2017). Tumor evolution: Linear, branching, neutral or punctuated?. Biochim. Biophys. Acta Rev. Cancer.

[14] Meacham CE, Morrison SJ (2013). Tumour heterogeneity and cancer cell plasticity. Nature.

[15] Marx V (2014). Cell communication: stop the microbial chatter. Nature.

[16] Tabassum DP, Polyak K (2015). Tumorigenesis: it takes a village. Nat. Rev. Cancer.

[17] Inda M-M (2010). Tumor heterogeneity is an active process maintained by a mutant EGFR-induced cytokine circuit in glioblastoma. Genes Dev..

[18] Calbo J (2011). A functional role for tumor cell heterogeneity in a mouse model of small cell lung cancer. Cancer Cell.

[19] Chapman A (2014). Heterogeneous tumor subpopulations cooperate to drive invasion. Cell Rep..

[20] Cleary AS, Leonard TL, Gestl SA, Gunther EJ (2014). Tumour cell heterogeneity maintained by cooperating subclones in Wnt-driven mammary cancers. Nature.

[21] Hobor S (2014). TGFα and amphiregulin paracrine network promotes resistance to EGFR blockade in colorectal cancer cells. Clin. Cancer Res. Off. J. Am. Assoc. Cancer Res..

[22] Marusyk A (2014). Non-cell-autonomous driving of tumour growth supports sub-clonal heterogeneity. Nature.

[23] Archetti M, Ferraro DA, Christofori G (2015). Heterogeneity for IGF-II production maintained by public goods dynamics in neuroendocrine pancreatic cancer. Proc. Natl. Acad. Sci. USA.

[24] Morris LGT (2016). Pan-cancer analysis of intratumor heterogeneity as a prognostic determinant of survival. Oncotarget.

[25] Meyer M (2015). Single cell-derived clonal analysis of human glioblastoma links functional and genomic heterogeneity. Proc. Natl. Acad. Sci. USA.

[26] Segerman A (2016). Clonal Variation in Drug and Radiation Response among Glioma-Initiating Cells Is Linked to Proneural-Mesenchymal Transition. Cell Rep..

[27] Reinartz R (2017). Functional Subclone Profiling for Prediction of Treatment-Induced Intratumor Population Shifts and Discovery of Rational Drug Combinations in Human Glioblastoma. Clin. Cancer Res. Off. J. Am. Assoc. Cancer Res..

[28] Lan X (2017). Fate mapping of human glioblastoma reveals an invariant stem cell hierarchy. Nature.

[29] Akgül Seçkin, Patch Ann-Marie, D’Souza Rochelle C.J., Mukhopadhyay Pamela, Nones Katia, Kempe Sarah, Kazakoff Stephen H., Jeffree Rosalind L., Stringer Brett W, Pearson John V., Waddell Nicola, Day Bryan W. (2019). Intratumoural Heterogeneity Underlies Distinct Therapy Responses and Treatment Resistance in Glioblastoma. Cancers.

[30] Mardis ER (2015). Xenografts as Models of Clonal Selection and Acquired Resistance to Therapy. Clin. Chem..

[31] Cassidy JW, Caldas C, Bruna A (2015). Maintaining Tumor Heterogeneity in Patient-Derived Tumor Xenografts. Cancer Res..

[32] Konen J (2017). Image-guided genomics of phenotypically heterogeneous populations reveals vascular signalling during symbiotic collective cancer invasion. Nat. Commun..

[33] Haeger A, Wolf K, Zegers MM, Friedl P (2015). Collective cell migration: guidance principles and hierarchies. Trends Cell Biol..

[34] Kadari Amrita, Pooja Deep, Gora Ravuri Halley, Gudem Sagarika, Kolapalli Venkata Ramana Murthy, Kulhari Hitesh, Sistla Ramakrishna (2018). Design of multifunctional peptide collaborated and docetaxel loaded lipid nanoparticles for antiglioma therapy. European Journal of Pharmaceutics and Biopharmaceutics.

[35] Gallego-Yerga L (2017). Docetaxel-Loaded Nanoparticles Assembled from β-Cyclodextrin/Calixarene Giant Surfactants: Physicochemical Properties and Cytotoxic Effect in Prostate Cancer and Glioblastoma Cells. Front. Pharmacol..

[36] Coluccia D (2018). Enhancing glioblastoma treatment using cisplatin-gold-nanoparticle conjugates and targeted delivery with magnetic resonance-guided focused ultrasound. Nanomedicine Nanotechnol. Biol. Med..

[37] Allen M, Bjerke M, Edlund H, Nelander S, Westermark B (2016). Origin of the U87MG glioma cell line: Good news and bad news. Sci. Transl. Med..

[38] Bonavia R, Inda M-M, Cavenee WK, Furnari FB (2011). Heterogeneity Maintenance in Glioblastoma: A Social Network. Cancer Res..

[39] Zhou H, Neelakantan D, Ford HL (2017). Clonal cooperativity in heterogenous cancers. Semin. Cell Dev. Biol..

[40] Martincorena Iñigo, Fowler Joanna C., Wabik Agnieszka, Lawson Andrew R. J., Abascal Federico, Hall Michael W. J., Cagan Alex, Murai Kasumi, Mahbubani Krishnaa, Stratton Michael R., Fitzgerald Rebecca C., Handford Penny A., Campbell Peter J., Saeb-Parsy Kourosh, Jones Philip H. (2018). Somatic mutant clones colonize the human esophagus with age. Science.

[41] Friedrich J, Seidel C, Ebner R, Kunz-schughart LA (2009). Spheroid-based drug screen: considerations and practical approach. Nat. Protoc..

[42] Mueller C, Liotta LA, Espina V (2010). Reverse phase protein microarrays advance to use in clinical trials. Mol. Oncol..

[43] Chiechi A (2012). Improved data normalization methods for reverse phase protein microarray analysis of complex biological samples. BioTechniques.

[44] Popic V (2015). Fast and scalable inference of multi-sample cancer lineages. Genome Biol..

[45] R Core Team. *R: A Language and Environment for Statistical Computing*. (R Foundation for Statistical Computing, 2012).

[46] Szklarczyk D (2017). The STRING database in 2017: quality-controlled protein-protein association networks, made broadly accessible. Nucleic Acids Res..

[47] Bastian, M., Heymann, S. & Jacomy, M. Gephi: An Open Source Software for Exploring and Manipulating Networks. In *Third International AAAI Conference on Weblogs and Social Media* (2009).

[48] Lê S, Josse J, Husson F (2008). FactoMineR: An R Package for Multivariate Analysis. J. Stat. Softw..

