# Supplementary material for: A new model isolates glioblastoma clonal interactions and reveals unexpected modes for regulating motility, proliferation, and drug resistance
Source: Sci Rep. 2019 Nov 22;9:17380. doi: 10.1038/s41598-019-53850-7 (PMC6874607; doi:10.1038/s41598-019-53850-7)
Supplement: Supplementary file 1 — Supplementary Dataset 1 [file 41598_2019_53850_MOESM1_ESM.pdf]

A new model isolates glioblastoma clonal interactions and reveals unexpected modes for regulating motility, proliferation, and drug resistance

Justin B Davis[1], Sreshta S Krishna[1], Ryan Abi Jomaa[1], Cindy Duong[1], Virginia Espina[1], Lance A Liotta[1], Claudius Mueller[1*]

[1]Center for Applied Proteomics and Molecular Medicine, George Mason University, 10920 George Mason Circle, Manassas, VA 20110, USA

Supplemental Figure 1. A-D Highest and lowest (phospho)protein abundance quartiles for U87MG cell line clonal subtypes 2, 3, 4, and 6. Protein-protein interactions as predicted by STRING were plotted using Gephi, with radial arms representing separate protein interaction clusters and color shade corresponding to protein abundance level (proteins described by gene names as understood by STRING, dark red=high, dark blue=low).

A.

Subtype 2

(Clones: S, U, V, W)

high
protein level/phosphorylation

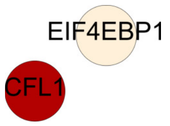

low
protein level/phosphorylation

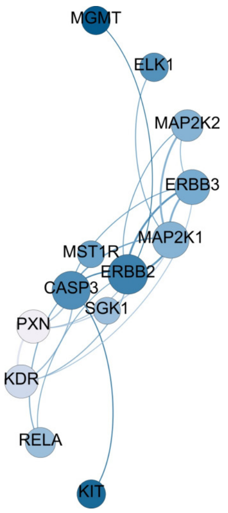

B.

Subtype 3
(Clones: H, K, M)

high
protein level/phosphorylation

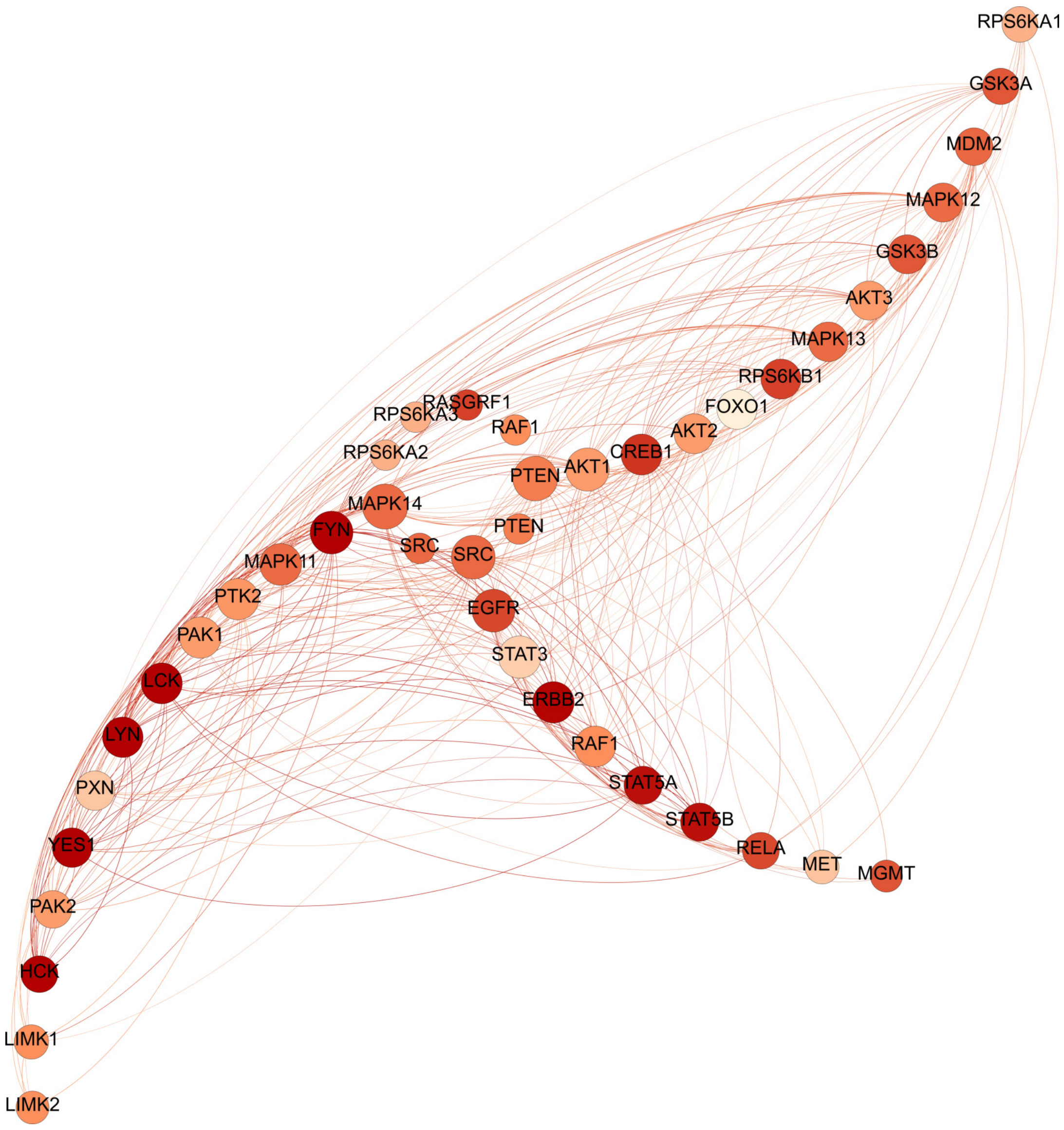

low
protein level/phosphorylation

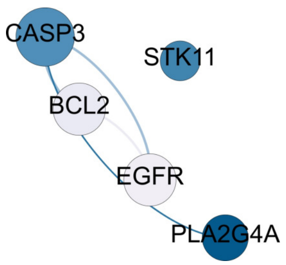

c.

Subtype 4

(Clones: F, G)

high
protein level/phosphorylation

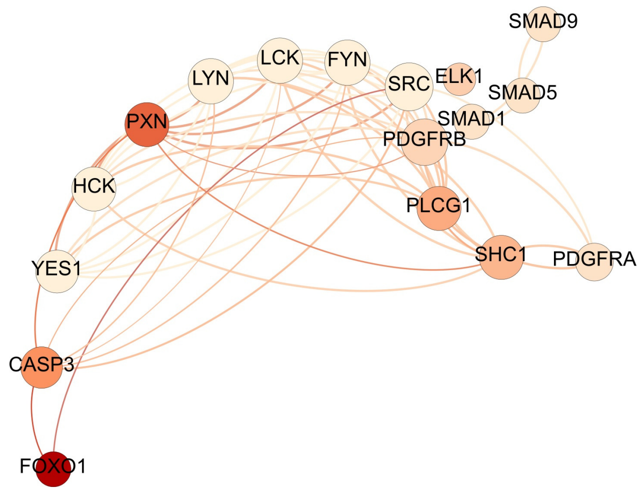

low
protein level/phosphorylation

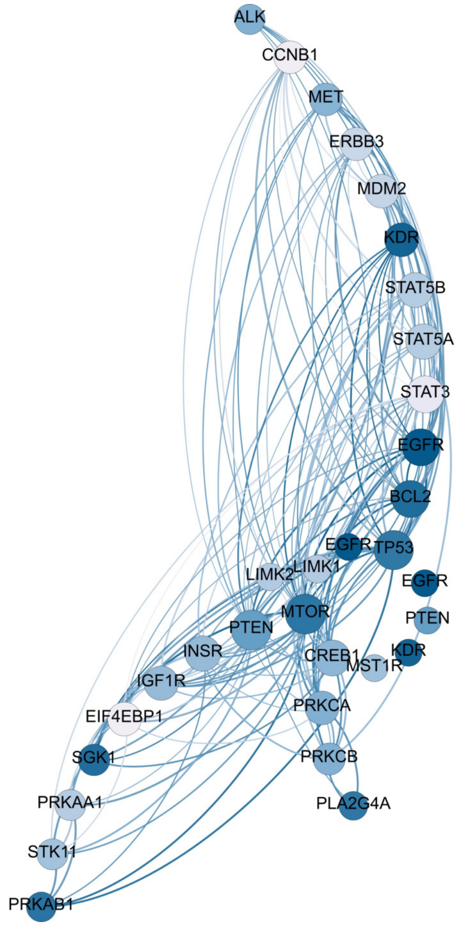

D.

Subtype 6

(Clones: P, R)

high
protein level/phosphorylation

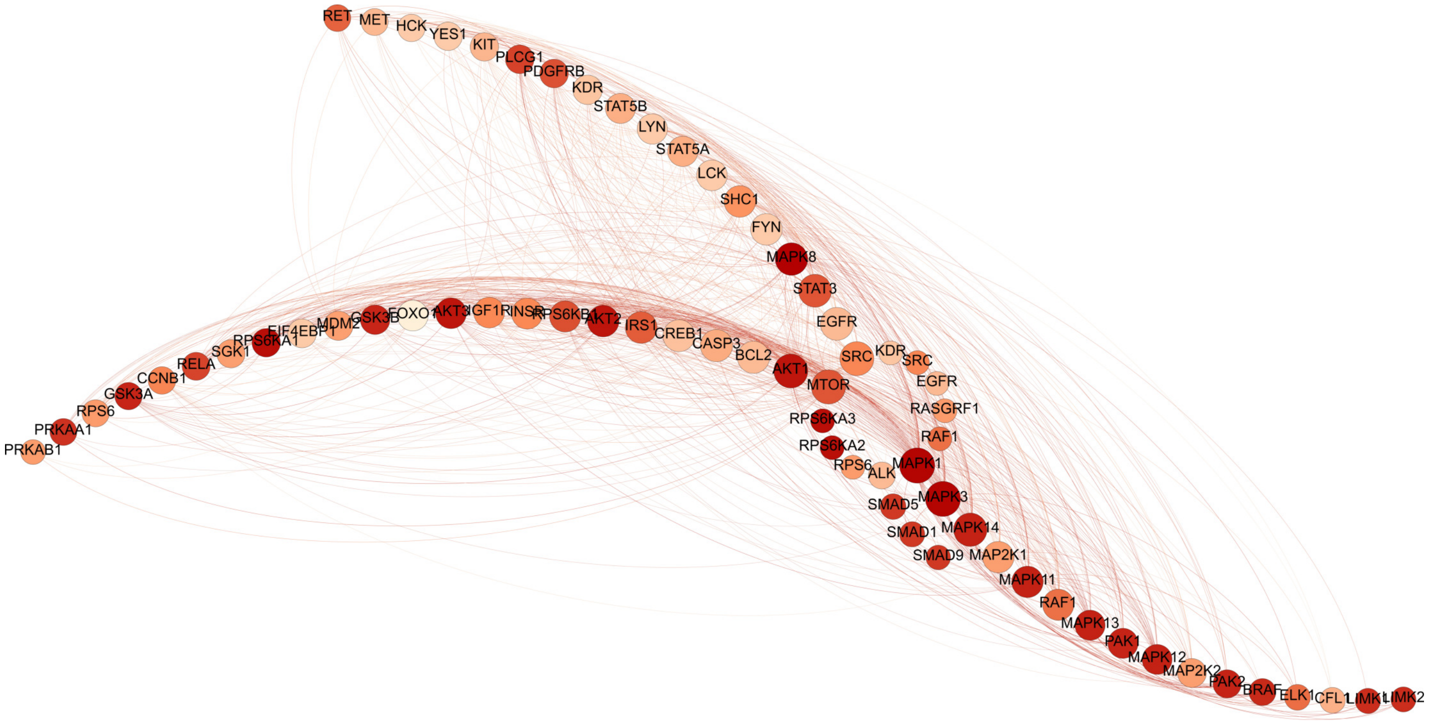

low
protein level/phosphorylation

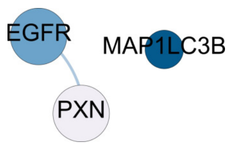

Supplemental Figure 2. Treatment response of clones C and E to cisplatin and docetaxel. To be able to assess clonal interactions at the critical transition from cytostatic to cytotoxic treatment response, we calculated the actual "growth rate" (cell doublings/day = log2(CellCountfinal/CellCountoriginal)/6 days) (A, B). This is in contrast to the often used "% cell survival", which does not allow differentiation of cytotoxic and cytostatic response (C, D). Drug concentrations closest to the cytostatic/cytotoxic response transition for at least one of the measured clones were chosen for further analysis.

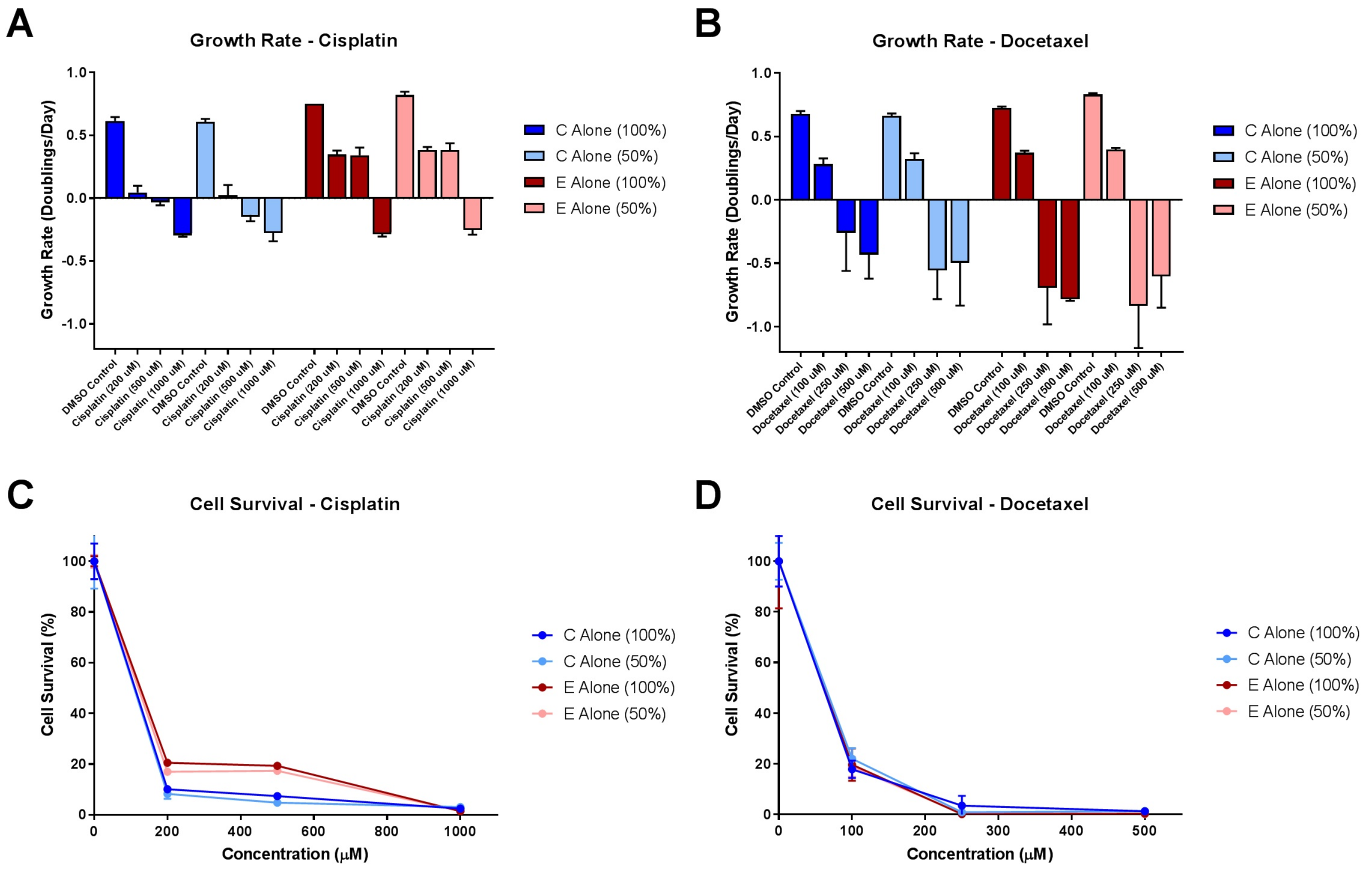

Supplemental Table 1: Antibodies used for reverse phase protein microarray analysis.

Antibody	Vendor & Catalog Number	Dilution
4E-BP1 Ser65	Cell Signaling Technology #9451	1:50
Akt Ser473	Cell Signaling Technology #9271	1:100
Akt Thr308	Cell Signaling Technology #9275	1:100
ALK Tyr1586	Cell Signaling Technology #3348	1:200
AMPKα1 Ser485	Cell Signaling Technology #4184	1:50
AMPKβ1 Ser108	Cell Signaling Technology #4181	1:50
B-Raf Ser445	Cell Signaling Technology #2696	1:50
Bcl2 Ser70	Cell Signaling Technology #2827	1:50
cKit Tyr703	Cell Signaling Technology #3073	1:50
Cleaved Caspase 3 Asp175	Cell Signaling Technology #9661	1:50
Cleaved PARP Asp214	Cell Signaling Technology #9541	1:100
cMet	Abcam #ab51067	1:200
Cofilin Ser3	Cell Signaling Technology #3313	1:100
cPLA2 Ser505	Cell Signaling Technology #2831	1:1000
cRAF Ser338	Cell Signaling Technology #9427	1:200
CREB Ser133	Cell Signaling Technology #9198	1:100
Cyclin B1	Cell Signaling Technology #4135	1:200
E-Cadherin	Cell Signaling Technology #4065	1:100
EGFR	Cell Signaling Technology #2232	1:100
EGFR Tyr1068	Cell Signaling Technology #2234	1:50
EGFR Tyr1148	BioSource #44-792	1:100
EGFR Tyr1173	BioSource #44-794	1:100
Elk1 Ser383	Cell Signaling Technology #9181	1:100
ErbB2 Tyr1248	Cell Signaling Technology #2247	1:100
ErbB3 Tyr1289	Cell Signaling Technology #4791	1:200
ERK Thr202/Tyr204	Cell Signaling Technology #9101	1:1000
FAK Tyr576/Tyr577	Cell Signaling Technology #3281	1:200
FKHR Ser256	Cell Signaling Technology #9461	1:100
GSK3α/β Ser21/Ser9	Cell Signaling Technology #9331	1:100
IGF1R Tyr1135/Tyr1136, IR Tyr1150/Tyr1151	Cell Signaling Technology #3024	1:200
IRS1 Ser612	Cell Signaling Technology #2386	1:200
JNK Thr183/Tyr185	Cell Signaling Technology #9251	1:100
LC3B	Cell Signaling Technology #2775	1:100
LIMK1/2 Thr508/Thr505	Cell Signaling Technology #3841	1:100
LKB1 Ser334	Cell Signaling Technology #3055	1:50
MDM2 Ser166	Cell Signaling Technology #3521	1:100
MEK1/2 Ser217/Ser221	Cell Signaling Technology #9121	1:500
Met Tyr1234/Tyr1235	Cell Signaling Technology #3126	1:200
MGMT	Cell Signaling Technology #2739	1:2000
mTOR Ser2448	Cell Signaling Technology #2971	1:100
NfκB p65 Ser536	Cell Signaling Technology #3031	1:100
p38 Thr180/Tyr182	Cell Signaling Technology #9211	1:100
p53	Cell Signaling Technology #9282	1:1000
p70 S6 Kinase Thr412	Upstate #07-018	1:500
p90RSK Ser380	Cell Signaling Technology #9341	1:200
PAK1/2 Ser199/Ser204/Ser192/Ser197	Cell Signaling Technology #2605	1:50
Paxilin Tyr118	Cell Signaling Technology #2541	1:500
PDGFRα Tyr754	Cell Signaling Technology #2992	1:500
PDGFRβ Tyr716	Upstate #07-021	1:200

Supplemental Table 2: Short Tandem Repeat (STR) profiling authenticates clones B, C, D, and E as of U87MG origin. While the Y allele of amelogenin was not matched, Y-chromosome painting and Q-band assay confirmed that the clones were male in origin.

% Match to HTB-14	ATCC ® Cat. No.	Designation	D5S818	D13S317	D7S820	D16S539	vWA	THO1	AMEL	TPOX	CSF1PO
100%	HTB-14 (Reference Database Profile)	U-87 MG; Glioblastoma-Astrocytoma; Human (Homo sapiens)	11,12	8,11	8,9	12	15,17	9.3	X,Y	8	10,11
93%	CLONE B	U87MG	11,12	8,11	8,9	12	15,17	9.3	X	8	10,11
93%	CLONE C	U87MG	11,12	8,11	8,9	12	15,17	9.3	X	8	10,11
93%	CLONE D	U87MG	11,12	8,11	8,9	12	15,17	9.3	X	8	10,11
93%	CLONE E	U87MG	11,12	8,11	8,9	12	15,17	9.3	X	8	10,11
